# Microstructural Study of MgB_2_ in the LiBH_4_-MgH_2_ Composite by Using TEM

**DOI:** 10.3390/nano12111893

**Published:** 2022-05-31

**Authors:** Ou Jin, Yuanyuan Shang, Xiaohui Huang, Xiaoke Mu, Dorothée Vinga Szabó, Thi Thu Le, Stefan Wagner, Christian Kübel, Claudio Pistidda, Astrid Pundt

**Affiliations:** 1 Institute of Applied Materials, Karlsruhe Institute of Technology, 76131 Karlsruhe, Germany; ou.jin@kit.edu (O.J.); dorothee.szabo@kit.edu (D.V.S.); stefan.wagner3@kit.edu (S.W.); 2 Institute of Nanotechnology, Karlsruhe Institute of Technology, 76344 Eggenstein-Leopoldshafen, Germany; xiaohui.huang@partner.kit.edu (X.H.); xiaoke.mu@kit.edu (X.M.); christian.kuebel@kit.edu (C.K.); 3 Institute of Hydrogen Technology, Helmholtz-Zentrum Hereon GmbH, 21502 Geesthacht, Germany; yuanyuan.shang@hzg.de (Y.S.); thi.le@hzg.de (T.T.L.); claudio.pistidda@hzg.de (C.P.); 4 Karlsruhe Nano Micro Facility, Karlsruhe Institute of Technology, 76344 Eggenstein-Leopoldshafen, Germany; 5 Joint Research Laboratory Nanomaterials, Technical University of Darmstadt, 64206 Darmstadt, Germany

**Keywords:** hydrogen storage, transmission electron microscopy, crystallography, reactive hydride composite, additive

## Abstract

The hampered kinetics of reactive hydride composites (RHCs) in hydrogen storage and release, which limits their use for extensive applications in hydrogen storage S1and energy conversion, can be improved using additives. However, the mechanism of the kinetic restriction and the additive effect on promoting the kinetics have remained unclear. These uncertainties are addressed by utilizing versatile transmission electron microscopy (TEM) on the LiBH_4_-MgH_2_ composite under the influence of the 3TiCl_3_·AlCl_3_ additives. The formation of the MgB_2_ phase, as the rate-limiting step, is emphatically studied. According to the observations, the heterogeneous nucleation of MgB_2_ relies on different nucleation centers (Mg or TiB_2_ and AlB_2_). The varied nucleation and growth of MgB_2_ are related to the in-plane strain energy density at the interface, resulting from the atomic misfit between MgB_2_ and its nucleation centers. This leads to distinct MgB_2_ morphologies (bars and platelets) and different performances in the dehydrogenation kinetics of LiBH_4_-MgH_2_. It was found that the formation of numerous MgB_2_ platelets is regarded as the origin of the kinetic improvement. Therefore, to promote dehydrogenation kinetics in comparable RHC systems for hydrogen storage, it is suggested to select additives delivering a small atomic misfit.

## 1. Introduction

Hydrogen is a clean and reproducible energy carrier with the highest gravimetric energy density of ~120 kJ g^−1^. For extensive applications of hydrogen, advanced hydrogen storage materials are demanded to store hydrogen safely and efficiently. Reactive hydride composites (RHCs) have been studied intensively due to their exceptionally reversible hydrogen storage capacity [1]. These materials were initially derived from light metal complex hydrides (e.g., LiBH_4_, LiNH_2_, NaAlH_4_, etc.) in combination with a second hydride (e.g., LiH, MgH_2_, etc.) [2,3,4]. Among various RHCs, the LiBH_4_-MgH_2_ composite is one of the most competitive candidates for both on- and off-board applications, based on the International Energy Agency Task 22 [5]. According to prior studies, the related decomposition reaction occurs in two steps [6]:

2LiBH_4_ + MgH_2_ → 2LiBH_4_ + Mg + H_2_ → 2LiH + MgB_2_ +4H_2_ (1)

Compared with the hydrogen capacity of ~18.5 wt% in pristine LiBH_4_, about 11.4 wt% of hydrogen can still be yielded with the LiBH_4_-MgH_2_ composite, while the thermodynamic properties are significantly improved by the addition of MgH_2_, resulting in overall superior performance in hydrogen storage. For LiBH_4_ alone, the standard enthalpy of decomposition is about 70 kJ mol^−1^ H_2_ [7]. This value corresponds to a decomposition temperature of about 400 °C under atmospheric pressure [7,8,9]. For the LiBH_4_-MgH_2_ composite, the standard decomposition enthalpy is reduced to about 45 kJ mol^−1^ H_2_ [9,10], which is ascribed to the exothermic formation of MgB_2_ during the endothermic two-step decomposition process of the LiBH_4_-MgH_2_ composite (see Equation (1)) [3,10]. This results in a notable reduction in the decomposition temperature down to about 170 °C under atmospheric pressure [4,9].

However, in contrast to the thermodynamic predictions, the decomposition of the LiBH_4_-MgH_2_ composite for hydrogen release is kinetically limited and barely triggered at moderate temperatures [4,6]. Bösenberg et al. ascribed the sluggish kinetic behavior to the nucleation of MgB_2_ as the rate-limiting step during dehydrogenation [11,12]. This is prominently reflected in the long incubation period, after the complete decomposition of MgH_2_ into Mg (the first dehydrogenation step in Equation (1)) and before the massive decomposition of LiBH_4_ (the second dehydrogenation step in Equation (1)).

Current research on enhancing the kinetics of hydride compounds mainly focuses on nanoconfinement using various carbon scaffolds and on utilizing transition metal-based additives [13,14,15,16]. The additives, in particular, provide an impressive boost to the dehydrogenation kinetics of RHCs, with their hydrogen storage capacities being well-preserved. As for the LiBH_4_-MgH_2_ composite, it has been reported that the enhanced kinetics can be attributed to a significant promotion of the heterogeneous nucleation of MgB_2_ by additives [17,18]. However, their role in the decomposition path has not yet been explicitly understood due to a lack of microscopic investigations; thus, the mechanism of MgB_2_ nucleation and growth in terms of crystallography has remained vague [11]. Complementing the missing knowledge is essential for understanding the reaction process behind the kinetic improvement of the material.

In this study, the additive effect on MgB_2_ nucleation and growth in the LiBH_4_-MgH_2_ composite is clarified by determining the MgB_2_ morphology and its crystallographic orientations toward nucleation centers. Transmission electron microscopy (TEM) studies were carried out on the microstructural evolution of MgB_2_ in samples with and without Ti- and Al-based additives using a very high content. This allowed us to reveal the details of how the microstructural boundary conditions determine the decomposition kinetics of the system.

## 2. Experimental Section

### 2.1. Material Preparations

The reactants were provided in powder form by the following commercial suppliers: MgH_2_ (95% purity) from Rockwood Lithium GmbH, LiBH_4_ (95% purity) from Sigma-Aldrich, and 3TiCl_3_·AlCl_3_ (about 76–78% purity) from Fischer Scientific. The LiBH_4_-MgH_2_ composite was prepared with a molar content of x% 3TiCl_3_·AlCl_3_ (x = 0, 0.625, and 20). The large additive content of 20 mol% is chosen to maximize the additive effect on the MgB_2_ morphology. To achieve a fine mixing of the reactants and an even dispersion of the additives, the prepared material mixtures (3 g)—namely, 2LiBH_4_-MgH_2_ or 2LiBH_4_-MgH_2_-3TiCl_3_·AlCl_3_—were charged into stainless-steel vials with stainless steel balls in a ball to powder ratio of 20:1. The milling proceeded for 400 min using a Spex 8000 M Mixer Mill. Both the powder handling and milling were always performed under an argon atmosphere in a glovebox (O_2_, H_2_O < 0.5 ppm).

### 2.2. Kinetic Measurements

The volumetric measurements were performed using a custom-built Sievert’s-type apparatus. The milled sample (~170 mg) was charged into the stainless-steel sample holder of the measuring apparatus. The samples were annealed from room temperature to 400 °C at a heating rate of 10 °C min^−1^ under a hydrogen atmosphere of 4 bar. After reaching the target temperature of 400 °C, the materials were kept under isothermal conditions for several hours.

### 2.3. XRD Experiments

The ex situ XRD experiments were based on a Bruker D8 Discover diffractometer equipped with a Cu X-ray source (λ = 1.54184 Å) and a 2D VANTEC detector. The operating voltage and current were 50 kV and 1000 mA. The diffraction patterns were acquired in the 2θ range from 10° to 90° with a step size of 0.005°, Δ2θ = 10°, and the exposure time for each step of 400 s. To prevent oxidation phenomena during the acquirement of the XRD pattern, the specimens were sealed in an argon-filled sample holder made of poly (methyl methacrylate) (PMMA).

### 2.4. TEM Experiments

TEM experiments were performed using a Themis-Z 60-300 (Thermo Fisher Scientific Inc., Waltham, MA, USA) equipped with a monochromator and double aberration correctors (probe and image Cs correctors), operated at 300 kV. TEM sample preparation was carried out under an argon atmosphere in a glovebox (O_2_, H_2_O < 0.5 ppm). Sample powders were dispersed in toluene and ultra-sonicated for 1 min before being distributed on lacey-carbon coated gold TEM grids S166-A3-V (Ted Pella Inc., Redding, CA, USA). Subsequently, they were transferred under argon from the glovebox into the microscope with a vacuum transfer holder 648 (Gatan Inc., Pleasanton, CA, USA).

The beam current varied from 50 to 100 pA throughout all TEM experiments. Selected area electron diffraction (SAED) patterns, TEM, and high-resolution TEM (HRTEM) images were collected using a OneView camera (Gatan Inc., Pleasanton, CA, USA). Scanning TEM (STEM) images were recorded via a high-angle annular dark-field (HAADF) detector with a convergence angle of 21.5 mrad and a camera length of 93 mm. Energy-dispersive X-ray spectroscopy (EDX) spectrum-imaging (SI) was executed with a Super-X windowless EDX detector (Thermo Fisher Scientific Inc., Waltham, MA, USA) using the same parameters as in STEM mode.

Electron tomography was carried out using a Fischione 2020 tomography holder in STEM mode with the same STEM parameters as mentioned above. HAADF-STEM Tilt series with image dimensions of 2048 × 2048 pixels were collected using Xplore3D (Thermo Fisher Scientific) over a tilt range in increments of 2° from −72° to 78° for the desorbed 2LiBH_4_-MgH_2_ without additives and from −72° to 68° for the desorbed 2LiBH_4_-MgH_2_ with 20 mol% 3TiCl_3_·AlCl_3_. The alignment of the tilt series was performed by IMOD using 10 nm gold colloidal particles as fiducial markers, which leads to the respective mean residual error of 0.736 and 0.129 voxels [19]. The aligned tilt series were then reconstructed using the algorithm simultaneous iterative reconstruction technique (SIRT) with 100 iterations by Inspect3D (Thermo Fisher Scientific) [20]. The 3D visualizations were realized by Avizo 2020.2 (Thermo Fisher Scientific).

In addition, 4D-STEM measurements were carried out in µ probe mode using the OneView camera, with a convergence angle of 0.47 mrad, a camera length of 580 mm, and an acquisition time of 60 ms for each diffraction pattern, and a dose of ~1.5 × 10^5^ e nm^−2^. STEM electron energy-loss spectroscopy (EELS) SI was acquired using a Gatan Continuum 970 HighRes imaging filter (GIF) (Gatan Inc., Pleasanton, CA, USA) in dual-EELS mode with 5 ms acquisition time for each low-loss spectrum, 20 ms for each high-loss spectrum, 21.5 mrad convergence angle, 40 mrad collection angle, and 0.3 eV per channel energy dispersion, leading to a measured energy spread of 2.0 eV on the camera. Both the EDX SI and EELS SI data were denoised via principal component analysis (PCA), which effectively reduces the random noise generated during the signal recording [21,22].

## 3. Results and Discussion

### 3.1. Material Characterization via XRD and Kinetic Performance

**Figure 1:**
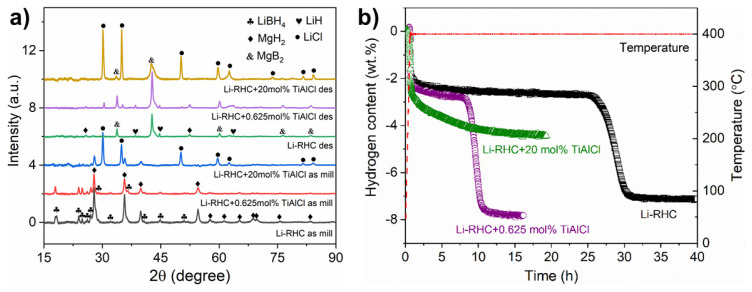
(**a**) XRD patterns of 2LiBH_4_-MgH_2_ with x mol% 3TiCl_3_·AlCl_3_ (x = 0, 0.625, and 20) after milling and after desorption; (**b**) desorption kinetics of 2LiBH_4_-MgH_2_ with x mol% 3TiCl_3_·AlCl_3_ (x = 0, 0.625, and 20) at 400 °C and under 4 bar H_2_.

Figure 1a shows the X-ray diffraction (XRD) results of the system 2LiBH_4_-MgH_2_ with x mol% 3TiCl_3_·AlCl_3_ (x = 0, 0.625, and 20) in two different states: as-milled and after desorption. In general, no significant variation is visible for the sample without additives and the one containing 0.625 mol% 3TiCl_3_·AlCl_3_. However, after increasing the additive content to a high value of 20 mol%, the LiBH_4_ peaks for the as-milled state and the MgB_2_ peaks for the after-desorption state noticeably weakened, whereas strong LiCl peaks appeared for both states. This difference can be attributed to the reaction between LiBH_4_ and 3TiCl_3_·AlCl_3,_ which consumed a significant amount of LiBH_4_ that is responsible for the generation of MgB_2_ [18].

The kinetic performance of the corresponding materials is visualized in Figure 1b. In comparison with the pristine material, the incubation period was reduced from ~25 h to ~8 h with an additive content of 0.625 mol%. By adding 20 mol% 3TiCl_3_·AlCl_3_, the incubation stage disappeared, and the kinetics of the second desorption step for hydrogen release changed. The reduced hydrogen storage capacity of ~5 wt% for the 20 mol% 3TiCl_3_·AlCl_3_ sample is due to the significant consumption of LiBH_4_ during the reaction with additives.

### 3.2. MgB_2_ Formation without the Influence of Additives

**Figure 2:**
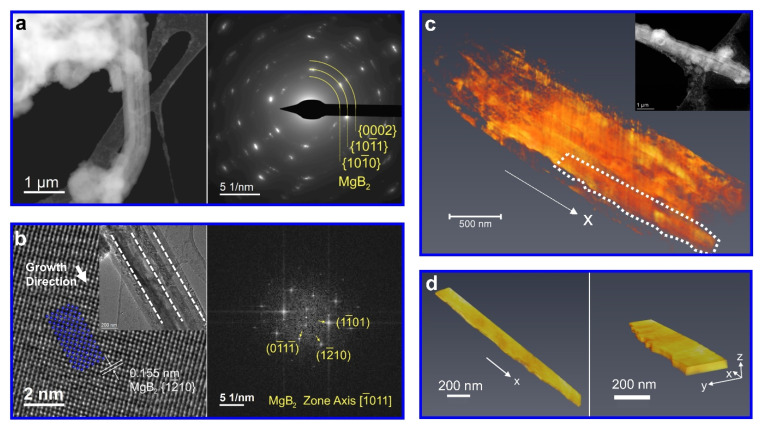
The results of 2LiBH_4_-MgH_2_ without additives after desorption: (**a**) STEM-HAADF image and the corresponding electron diffraction pattern; (**b**) HRTEM image with an inset of the local zoomed-out overview showing the growth direction of MgB_2_ bars, and the corresponding FFT; (**c**) volume rendering from tomographic reconstruction of MgB_2_ bars with an inset showing the corresponding STEM-HAADF image; (**d**) volume rendering of one selected MgB_2_ bar chosen from (**c**).

Figure 2a presents the morphology of the decomposed LiBH_4_-MgH_2_ composite—namely, LiH and MgB_2_. The local electron diffraction pattern indicates the existence of MgB_2_ crystals with a hexagonal closed packed (hcp) structure (P6m/mm, No. 191). The absence of crystallographic information on LiH is likely due to its instability under electron illumination and the low scattering power of Li and H. One impressive feature in Figure 2a is the bar-like morphology of crystals with a parallel arrangement. They were identified as MgB_2_ by HRTEM (Figure 2b), its fast Fourier transform (FFT) pattern, and the EDXS elemental map of Mg acquired from the corresponding area in Figure 2a (see Figure S1). The primary growth direction of MgB_2_ bars is thus determined to be ${\left[1\bar{2}10\right]}_{{\mathrm{MgB}}_{2}}$, which is along the long axis of a MgB_2_ bar (the inset in Figure 2b).

For a more comprehensive understanding of the MgB_2_ morphology, electron tomography was conducted. Figure 2c shows a volume rendering of MgB_2_ bars, reconstructed from the STEM-HAADF tomographic data, and Figure 2d exhibits a single MgB_2_ bar extracted from the selected region in Figure 2c. It should be noted that MgB_2_ bars possess a rectangular shape. This can be understood in combination with the study by Lee et al., claiming that a growth constraint exists for MgB_2_ along the c-axis ${\left[0002\right]}_{{\mathrm{MgB}}_{2}}$ [23], which is consistent with our investigation, as the constrained direction $\;{\left[0002\right]}_{{\mathrm{MgB}}_{2}}$ (z-axis in Figure 2d) lays perpendicular to the observed growth direction ${\left[1\bar{2}10\right]}_{{\mathrm{MgB}}_{2}}\;$(x-axis). Given the rectangular MgB_2_ bars, another growing direction along the y-axis corresponds to ${\left[10\bar{1}0\right]}_{{\mathrm{MgB}}_{2}}$, along which the growth is also limited to some extent. This is discussed in Section 3.5.

The observation of parallel MgB_2_ bars in Figure 2a implies that their nucleation and growth initially proceeded from the same class of atomic layers of one crystalline nucleation center. Given the two-step decomposition of the LiBH_4_-MgH_2_ composite (Equation (1)), Mg and LiBH_4_ are candidates for this nucleation center. Since LiBH_4_ has a melting point of approximately 275 °C and, therefore, remains in the liquid state during the desorption at 400 °C, Mg has to be the nucleation center for the heterogeneous nucleation of these MgB_2_ bars. Another requirement for the nucleation center is that it has to provide a sufficiently large superficial plane to nucleate several MgB_2_ bars with a lateral size of about 200 nm (Figure 2d). The observed Mg grains are large enough, with a size up to about 1 μm to meet this requirement (see Figure S2).

### 3.3. MgB_2_ Formation under the Influence of Additives

**Figure 3:**
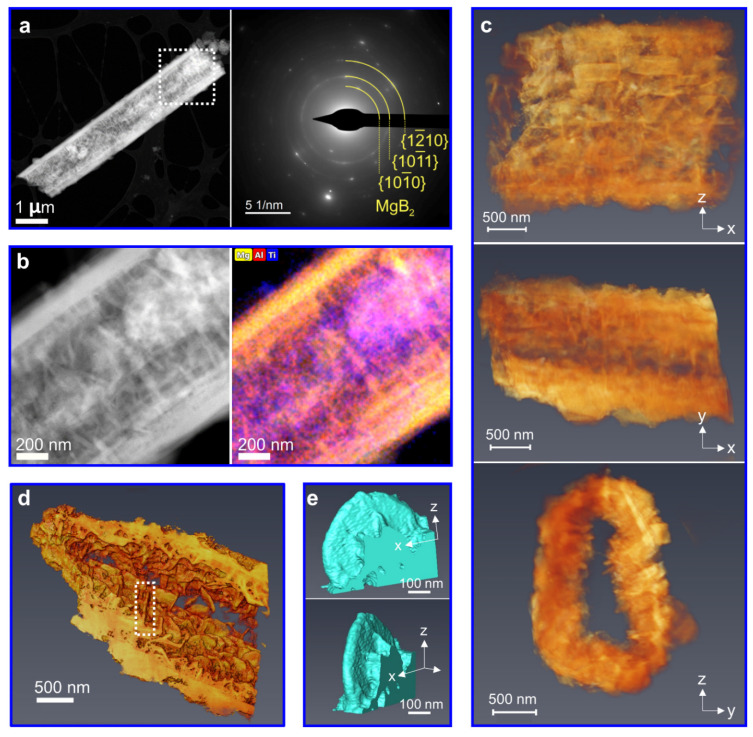
The results of 2LiBH_4_-MgH_2_ with 20 mol% 3TiCl_3_·AlCl_3_ after desorption: (**a**) STEM-HAADF image and the corresponding electron diffraction pattern; (**b**) STEM-HAADF image of the selected area in (**a**) and EDXS elemental map of Mg (yellow), Ti (blue), and Al (red); (**c**) volume rendering from tomographic reconstruction of a MgB_2_ hollow tube viewed at different angles; (**d**) surface rendering of the cross-section of (**c**); (**e**) surface rendering of a segmented MgB_2_ platelet selected from (**d**).

To reveal the additive’s effect on MgB_2_ nucleation and growth, an overdose of 20 mol% 3TiCl_3_·AlCl_3_ was taken for the LiBH_4_-MgH_2_ composite. Figure 3a shows the material morphology after desorption. According to the XRD result (Figure 1a) and the local electron diffraction pattern (Figure 3a), the element Mg exists only as MgB_2_. Therefore, the Mg EDX map (Figure 3b) can be directly regarded as representing the distribution of MgB_2_, which is comparable with the bright contrast in the HAADF image (Figure 3b). In general, the MgB_2_ morphology changed significantly, compared with what was previously reported in Section 3.2.

Figure 3c exhibits a reconstructed volume rendering of MgB_2_ in different directions. In this case, two different MgB_2_ morphologies can be distinguished. Orienting along the x-axis, the parallel-lying MgB_2_ bars grow into a hollow tube. Inside the tube, the second morphology of platelet-like MgB_2_ can be identified. It can be seen in Figure 3d that these MgB_2_ platelets grow from the tube walls of MgB_2_ bars in a more or less random orientation. For a closer look at these MgB_2_ platelets, one piece was cut out from Figure 3d and displayed as a segmented surface rendering in Figure 3e. In contrast to the rectangular shape of the MgB_2_ bars, these MgB_2_ platelets with a lateral size of about 500 nm possess a more irregular or semi-circular shape. In other words, no primary growth direction is observed for the MgB_2_ platelets. In addition, the MgB_2_ morphology of the sample with 0.625 mol% 3TiCl_3_·AlCl_3_ can be found in Figure S3 for comparison.

The platelet-like MgB_2_ morphology hints at another nucleation center that facilitates the formation of the MgB_2_ platelets. We suggest that this nucleation center stems from the additives, so the identification and location of these additives is another point of interest. As reported by T.-T. Le et al., the additive 3TiCl_3_·AlCl_3_ can react with LiBH_4_ and generate either AlTi_3_ or TiB_2_ and AlB_2_ [18]. Accordingly, rather than the initial additive 3TiCl_3_·AlCl_3_, these reaction products are considered to be the heterogeneous nucleation centers for the MgB_2_ platelets. Based on the STEM-EDX map in Figure 3b, both Ti and Al are dispersed inside the MgB_2_ tube, which is also indicative of their role as the nucleation center (also see Figure S4).

**Figure 4:**
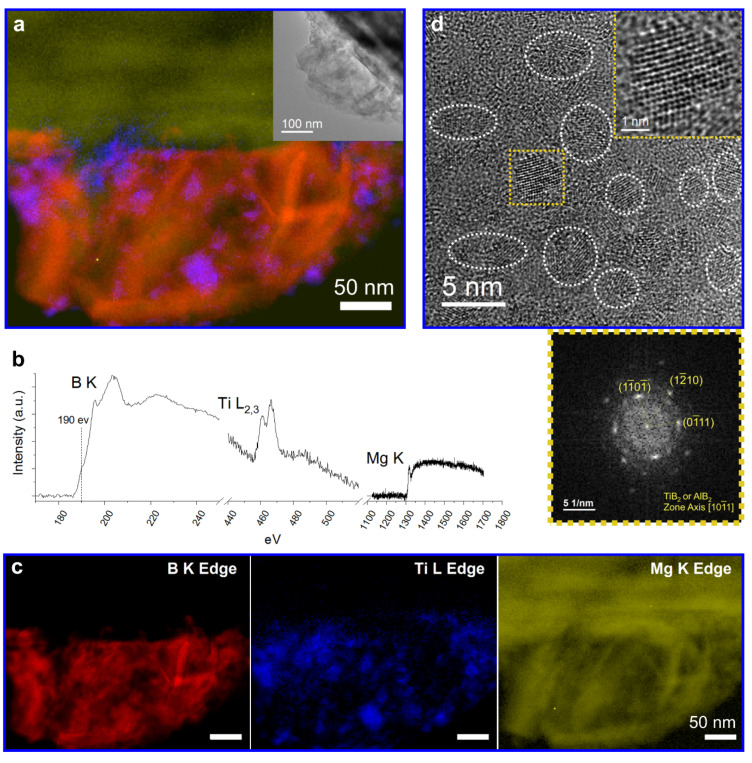
The results of 2LiBH_4_-MgH_2_ with 20 mol% 3TiCl_3_·AlCl_3_: (**a**) summed EELS elemental map, based on the background-subtracted EEL spectrum (**b**), and comprising the elemental distribution of B K-edge (red), Ti L_2,3_-edge (blue), and Mg K-edge (yellow) (**c**). The inset of a TEM bright-field image was recorded in the same area. In (**a**), the orange color is coming from the overlap between yellow and red, and is thus indicative of the correlation between Mg and B. Similarly, the color purple stands for the correlation between Ti and B. (**d**) HRTEM image of TiB_2_ (and AlB_2_) nanoparticles just after milling and the corresponding FFT of the inset showing the lattice of a single-crystalline TiB_2_ particle.

For further studies, the EELS SI was acquired. Figure 4a displays the summed elemental map based on the EEL spectrum in Figure 4b, comprising B K-edge in red, Ti L_2,3_-edge in blue, and Mg K-edge in yellow (Figure 4c). Therefore, the orange platelets that result from an overlap between Mg (yellow) and B (red) indicate the elemental correlation between Mg and B and suggest the location of MgB_2_. Furthermore, the background-subtracted B K-edge recorded in the orange area exhibits a pre-peak at about 190 eV (Figure 4b). This fine structure is evidence of a high and unfilled p-like density of states of Boron, which indicates the bonding between Mg and B and verifies the existence of MgB_2_ [24].

Similarly, the purple agglomerates in Figure 4a represent the spatial elemental correlation between Ti (blue) and B (red), which disperse around the orange MgB_2_ platelets. According to the HRTEM image and its FFT pattern (Figure 4d), the presence of TiB_2_ rather than AlTi_3_ can be confirmed already after the ball milling process. The HRTEM image corresponding to the purple agglomerates in Figure 4a can be found in Figure S5, which shows a further growth of TiB_2_ particles up to ~20 nm after desorption likely due to Ostwald ripening. The beneficial effect of TiB_2_ on accelerating the decomposition of the LiBH_4_-MgH_2_ composite was already reported by some studies, which also supports our characterizations [25,26]. Since AlB_2_ and TiB_2_ have the same space group (P6m/mm, No. 191), with almost the same lattice constants, AlB_2_ might have the same effect as TiB_2_, although they cannot be distinguished here.

**Figure 5:**
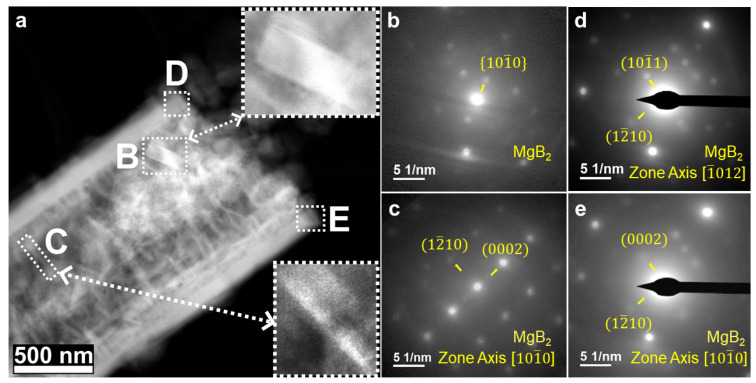
The results of 2LiBH_4_-MgH_2_ with 20 mol% 3TiCl_3_·AlCl_3_ after desorption: (**a**) STEM-HAADF image with insets of magnified areas B and C; (**b**–**e**) diffraction patterns acquired in the corresponding areas B–E in (**a**), which show the crystallographic orientation of MgB_2_ bars and platelets.

In Figure 5, the relationship between the crystallography and the morphology of the MgB_2_ bars and MgB_2_ platelets is revealed via 4D-STEM. Figure 5b–e provide local electron-diffraction patterns acquired from areas B-E in Figure 5a. A fragment of the MgB_2_ bar with a rectangular shape can be found in area B of Figure 5a, with the short axis along ${\left[10\bar{1}0\right]}_{{\mathrm{MgB}}_{2}}$ (Figure 5b). Figure 5d,e indicate the long axis direction of the MgB_2_ bars being along ${\left[1\bar{2}10\right]}_{{\mathrm{MgB}}_{2}}$. These investigated orientations are consistent with Figure 2. Thus, it can be summarized that the morphology of MgB_2_ bars with a rectangular shape has the long axis direction oriented along ${\left[1\bar{2}10\right]}_{{\mathrm{MgB}}_{2}}$, the short axis direction along ${\left[10\bar{1}0\right]}_{{\mathrm{MgB}}_{2}}$, and the thin direction along ${\left[0002\right]}_{{\mathrm{MgB}}_{2}}$. For MgB_2_ platelets, the thin direction of a platelet, which is also regarded as the growth-restricted direction, is along ${\left[0002\right]}_{{\mathrm{MgB}}_{2}}$ (Figure 5a,c). This growth restriction observed for more other MgB_2_ platelets can be seen in Figure S6.

### 3.4. Analysis of Orientation Relationships

Given the small size of TiB_2_ or/and AlB_2_ nanoparticles only up to about 20 nm (Figure S5) and the complexity of the overlapping phases, the interface between the nucleation centers and MgB_2_ cannot be investigated in the experiment. For this reason, their orientation relationships (ORs) cannot be experimentally determined in a conventional way by focusing on the interface. To determine the orientation relationship between the different nucleation centers and MgB_2_, we took advantage of the edge-to-edge matching model [27,28,29,30,31].

The edge-to-edge matching model builds on minimizing the energy of coherent interfaces between two adjacent materials, in this case, the nucleation center and the nucleating particle [27,30]. According to this model, the heterogeneous nucleation between the two phases is controlled by their interatomic misfit and their interplanar mismatch. The interatomic misfit is determined along the matching directions of the two phases. These matching directions are selected among their close or nearly close-packed directions identified by the linear atomic density. The interplanar mismatch (d-value mismatch) is determined between the matching planes, which are chosen from the close or nearly close-packed planes. As a rule of thumb, the interatomic misfit and the interplanar mismatch should generally not exceed 10% and 6%, which are regarded as the critical values [27]. On the other hand, minimizing the interatomic misfit has a higher priority than the d-value mismatch from an energetic perspective. A slight excess of the d-value mismatch above 6% is still acceptable, which can be subsequently refined by an additional rotation between the two phases about their matching directions [30].

Since MgB_2_, Mg, and TiB_2_ (and AlB_2_) have a similar hexagonal close-packed (hcp) crystal structure, their close or nearly close-packed directions and planes are similarly indexed. Based on their crystallographic characteristics, there are four possible close-packed directions $\langle 10\bar{1}0\rangle$, 〈0002〉, $\langle 1\bar{2}10\rangle$, and $\langle 1\bar{1}23\rangle$ and four possible close-packed planes $\left\{10\bar{1}0\right\}$, $\left\{10\bar{1}1\right\}$, {0002}, and $\left\{1\bar{2}10\right\}$ [29]. According to our observations, two distinct MgB_2_ morphologies were introduced:

1. Rectangular-shaped MgB_2_ bars that are constrained along ${\left[0002\right]}_{{\mathrm{MgB}}_{2}}$ and ${\left[10\bar{1}0\right]}_{{\mathrm{MgB}}_{2}}$, and primarily grow along ${\left[1\bar{2}10\right]}_{{\mathrm{MgB}}_{2}}$ from Mg grains (Figure 2 and Figure 5);
2. Semi-circular shaped MgB_2_ platelets that are constrained along ${\left[0002\right]}_{{\mathrm{MgB}}_{2}}$ and grow from TiB_2_ (and AlB_2_) nanoparticles (Figure 3, Figure 4 and Figure 5).

Given the large interatomic misfit of $\langle 0002\rangle {\;}_{{\mathrm{MgB}}_{2}}$, compared with the close-packed directions of Mg and TiB_2_ (and AlB_2_) (see Tables S1 and S2), ${\left\{0002\right\}}_{{\mathrm{MgB}}_{2}}$, as one of the close-packed planes, is supposed to be the matching plane for the heterogeneous nucleation of both MgB_2_ bars and MgB_2_ platelets on Mg and TiB_2_ (and AlB_2_).

For the MgB_2_ bars, the interplanar mismatch of ${\left\{0002\right\}}_{{\mathrm{MgB}}_{2}}$ concerning the possible matching plane of Mg is listed in Table 1. The only suitable matching planes with a reasonably low interplanar mismatch of ~8.5% are ${\left\{0002\right\}}_{{\mathrm{MgB}}_{2}}\;|\;{\left\{1\bar{2}10\right\}}_{\mathrm{Mg}}$. In addition, since the matching ${\left\{0002\right\}}_{{\mathrm{MgB}}_{2}}$ the plane should include the matching direction, only ${\langle 10\bar{1}0\rangle }_{{\mathrm{MgB}}_{2}}$ and ${\langle 1\bar{2}10\rangle }_{{\mathrm{MgB}}_{2}}$ fit for the matching direction. Similarly, only ${\langle 10\bar{1}0\rangle }_{\mathrm{Mg}}$ and ${\langle 0002\rangle }_{\mathrm{Mg}}$ are fitting for Mg, with ${\left\{1\bar{2}10\right\}}_{\mathrm{Mg}}$ being the matching plane. The resulting pairs of the matching directions with an interatomic misfit smaller than 10% turn out to be ${\langle 10\bar{1}0\rangle }_{{\mathrm{MgB}}_{2}}$||${\langle 10\bar{1}0\rangle }_{\mathrm{Mg}}$ and ${\langle 10\bar{1}0\rangle }_{{\mathrm{MgB}}_{2}}$||${\langle 0002\rangle }_{\mathrm{Mg}}$ (Table 2). For a refined matching, a tilting angle between ${\left\{0002\right\}}_{{\mathrm{MgB}}_{2}}$ and ${\left\{1\bar{2}10\right\}}_{\mathrm{Mg}}$ should also be considered (see Figure S7a,b). In summary, the possible orientation relationships between MgB_2_ and Mg are predicted to be as follows:

$${\langle 10\bar{1}0\rangle }_{{\mathrm{MgB}}_{2}}\;\left|\;\right|\;\;{\langle 10\bar{1}0\rangle }_{\mathrm{Mg}}\;\;\;\;{\left\{0002\right\}}_{{\mathrm{MgB}}_{2}}\;\sim \;2.0{}^{\circ}\;from\;\;{\left\{1\bar{2}10\right\}}_{\mathrm{Mg}}\;\;{\langle 10\bar{1}0\rangle }_{{\mathrm{MgB}}_{2}}\;\left|\;\right|\;\;{\langle 0002\rangle }_{\mathrm{Mg}}\;\;\;\;{\left\{0002\right\}}_{{\mathrm{MgB}}_{2}}\;\sim \;1.1{}^{\circ}\;from\;\;{\left\{1\bar{2}10\right\}}_{\mathrm{Mg}}$$

The orientation relationships between MgB_2_ and TiB_2_ (and/or AlB_2_) can be derived in the same way. The related interplanar mismatch is given in Table 3. In this case, it appears that ${\left\{0002\right\}}_{{\mathrm{MgB}}_{2}}$ pairing with ${\left\{0002\right\}}_{{\mathrm{TiB}}_{2}}$ (and/or ${\left\{0002\right\}}_{{\mathrm{AlB}}_{2}}$) results in the least mismatch.

According to Table 4, the possible matching direction are $\langle 10\bar{1}0\rangle$ and $\langle 1\bar{2}10\rangle$ for both MgB_2_ and TiB_2_ (and/or AlB_2_). The least interatomic misfit is thus obtained for the matching directions ${\langle 10\bar{1}0\rangle }_{{\mathrm{MgB}}_{2}}$||${\langle 10\bar{1}0\rangle }_{{\mathrm{TiB}}_{2\;}({\mathrm{AlB}}_{2})}$ and ${\langle 1\bar{2}10\rangle }_{{\mathrm{MgB}}_{2}}$||${\langle 1\bar{2}10\rangle }_{{\mathrm{TiB}}_{2\;}({\mathrm{AlB}}_{2})}$. Considering the rotation between the two matching planes (see Figure S7c,d), the possible orientation relationships between MgB_2_ and TiB_2_ (and/or AlB_2_) are as follows:

$${\langle 10\bar{1}0\rangle }_{{\mathrm{MgB}}_{2}}\;\left|\;\right|\;{\langle 10\bar{1}0\rangle }_{{\mathrm{TiB}}_{2\;}({\mathrm{AlB}}_{2})}\;\;\;\;{\left\{0002\right\}}_{{\mathrm{MgB}}_{2}}\;\sim \;0.03{}^{\circ}\;from\;\;{\left\{0002\right\}}_{{\mathrm{TiB}}_{2\;}({\mathrm{AlB}}_{2})}\;\;{\langle 1\bar{2}10\rangle }_{{\mathrm{MgB}}_{2}}\;\left|\;\right|\;{\langle 1\bar{2}10\rangle }_{{\mathrm{TiB}}_{2\;}({\mathrm{AlB}}_{2})}\;\;\;\;{\left\{0002\right\}}_{{\mathrm{MgB}}_{2}}\;\sim \;0.01{}^{\circ}\;from\;\;{\left\{0002\right\}}_{{\mathrm{TiB}}_{2\;}({\mathrm{AlB}}_{2})}\;$$

### 3.5. Varied Morphology and Kinetics

As predicted, the heterogeneous nucleation of MgB_2_ on Mg grains occurs along the directions ${\left[10\bar{1}0\right]}_{{\mathrm{MgB}}_{2}}$ and ${\left[0002\right]}_{{\mathrm{MgB}}_{2}}$. It is noted that the predicted orientation of MgB_2_ agrees with our experimental observations on the morphology of the MgB_2_ bars, as demonstrated in Figure 2 and Figure 5. The subsequent growth dominantly along ${\left[1\bar{2}10\right]}_{{\mathrm{MgB}}_{2}}$ is likely due to the constrained growth along ${\left[0002\right]}_{{\mathrm{MgB}}_{2}}$ as well as ${\left[10\bar{1}0\right]}_{{\mathrm{MgB}}_{2}}$, both of which are in-plane directions at the MgB_2_/Mg interface, resulting in a rectangular-shaped MgB_2_ bar. Conversely, the growth of the MgB_2_ platelets on TiB_2_ nanoparticles is only limited along ${\left[0002\right]}_{{\mathrm{MgB}}_{2}}$, one of the in-plane directions at the MgB_2_/TiB_2_ interface. According to Figure 3, these MgB_2_ platelets with a semi-circular shape are likely growing more randomly without a preferred growth direction.

The different MgB_2_ morphologies, ranging from MgB_2_ bars to MgB_2_ platelets, are here ascribed to the different extent of the strain energy density induced at the interface to the nucleation center. Different aspect ratios of $\left[1\bar{2}10\right]:\left[10\bar{1}0\right]:\left[0002\right]$ are determined to be about 50:5:1 for MgB_2_ bars growing on Mg (Figure 2d), and about 15:15:1 for MgB_2_ platelets growing on TiB_2_ (Figure 3e). As the elastic-strain energy density $\epsilon {\;\mathrm{is}\;\mathrm{proportional}\;\mathrm{to}\;Y\varepsilon }^{2}$, where Y is Young’s modulus of MgB_2_, and ε is the atomic misfit between MgB_2_ and Mg or TiB_2_, the strain energy density can be thus roughly estimated by the related misfits, by assuming constant Y for simplicity. Along the interatomic direction ${\left[10\bar{1}0\right]}_{{\mathrm{MgB}}_{2}}$, the induced strain energy density is approximated to be more than 6 times larger for MgB_2_ on Mg grains (misfit of 4.2%) than for MgB_2_ on TiB_2_ nanoparticles (misfit of 1.7%), as listed in Table 2 and Table 4. Thus, the MgB_2_ growth is hindered along the direction ${\left[10\bar{1}0\right]}_{{\mathrm{MgB}}_{2}}$ on Mg, leading to a lower aspect ratio $\left[10\bar{1}0\right]:\left[0002\right]$ of 5:1 for MgB_2_ growth on Mg, compared with 15:1 for MgB_2_ growth on TiB_2_. The compelling growth restriction along [0002] can be also well-interpreted by the large interplanar misfit up to ~8% in both cases (Table 1 and Table 3). Therefore, the difference in the elastic strain energy density here is considered to be the root of the different MgB_2_ morphologies, and furthermore, the changed desorption kinetic performances.

**Figure 6:**
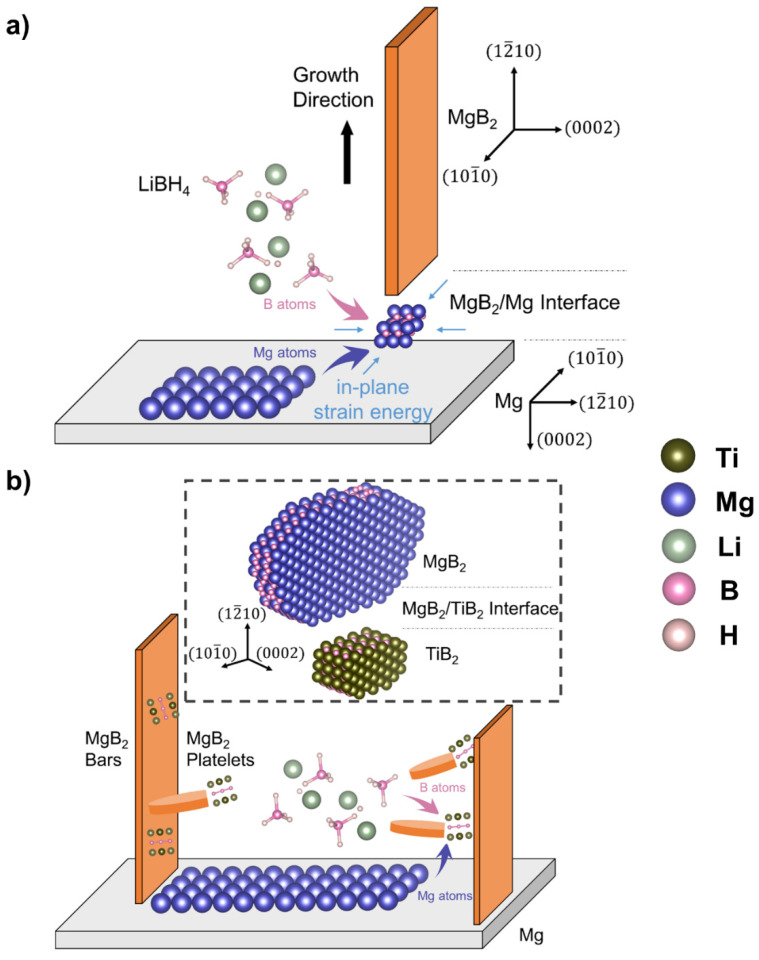
(**a**) Schematic illustration of how MgB_2_ bars are generated based on Mg grains following a certain crystallographic orientation relationship. The nucleation and growth of MgB_2_ may occur at the MgB_2_/Mg interface under control of the in-plane strain energy density; (**b**) schematic illustration of the nucleation and growth of MgB_2_ platelets based on TiB_2_ nanoparticles at the MgB_2_/TiB_2_ interface.

Figure 6 illustrates the complete process of the MgB_2_ formation as discussed above. For MgB_2_ growing on Mg grains, the further growth along ${\left[10\bar{1}0\right]}_{{\mathrm{MgB}}_{2}}$ is hampered due to a huge in-plane strain energy density, which forces itself to mainly grow in the out-of-plane direction ${\left[1\bar{2}10\right]}_{{\mathrm{MgB}}_{2}}$, ending up with these parallel-lying rectangular MgB_2_ bars (Figure 6a). On the contrary, with an interatomic misfit of 1.7% between MgB_2_ and TiB_2_ along ${\left[10\bar{1}0\right]}_{{\mathrm{MgB}}_{2}}$, this in-plane growth constraint is not relevant (Figure 6b). The further growth of MgB_2_ along the in-plane direction ${\left[10\bar{1}0\right]}_{{\mathrm{MgB}}_{2}}$, and the out-of-plane direction ${\left[1\bar{2}10\right]}_{{\mathrm{MgB}}_{2}}$ can thus continuously proceed, leading to semi-circular shaped MgB_2_ platelets. Furthermore, a critical value of the interatomic misfit between 1.7% and 4.2% may be expected, above which the in-plane growth of MgB_2_ is restricted. This may result in a deceleration of the MgB_2_ nucleation and growth and, therefore, a decelerated desorption kinetics for the LiBH_4_-MgH_2_ composite.

As the growth of MgB_2_ commonly leads to hexagonal plates [23], we speculate that the MgB_2_ formation occurs at the interface between MgB_2_ and its nucleation centers rather than on the MgB_2_ surface exposed to the liquefied LiBH_4_. This means that the formation of MgB_2_ is always under the control of the in-plane strain energy density (Figure 6). From this perspective, the highly regular rectangular shape of the MgB_2_ bars can be explained consistently. However, future in situ experiments are still required to confirm this speculation.

## 4. Conclusions

Manifold TEM studies on the LiBH_4_-MgH_2_ composite with and without additives, combined with the predictions of the edge-to-edge matching model on the orientation relationship between the nucleation center and the resulting MgB_2_ phase, contribute to a better understanding of the RHCs’ kinetics. According to this, the additives deliver a large number of nucleation centers with a small interatomic misfit of 1.7% to MgB_2_, corresponding to low in-plane strain energy density. They facilitate the nucleation and growth of MgB_2_, leading to the morphology of MgB_2_ semi-circular platelets. In contrast, large in-plane strain energy density is expected for the formation of MgB_2_ bars on Mg, limiting the growth of MgB_2_ and consequently slowing down the dehydrogenation kinetics. To further improve the kinetic performance of the LiBH_4_-MgH_2_ composite, we suggest that the atomic misfit delivered by additives with respect to MgB_2_ should be considered for future additive selection. It is also believed that these conclusions hold for other RHC systems interesting for hydrogen energy storage and release.

## Figures and Tables

**Table 1:** The interplanar misfit between MgB_2_ {0002} and possible match planes of Mg nucleation center (%).

MgB_2_/Mg	$\left\{0002\right\}|\left\{10\bar{1}1\right\}$	{0002}|{0002}	$\left\{0002\right\}|\left\{10\bar{1}0\right\}$	$\left\{0002\right\}|\left\{1\bar{2}10\right\}$
	39.8	48.6	58.4	8.5

**Table 2:** The interatomic misfit along possible matching directions between MgB_2_ and Mg nucleation center (%).

MgB_2_/Mg	$\langle 10\bar{1}0\rangle ||\langle 10\bar{1}0\rangle$	$\langle 10\bar{1}0\rangle ||\langle 0002\rangle$	$\langle 1\bar{2}10\rangle ||\langle 10\bar{1}0\rangle$	$\langle 1\bar{2}10\rangle ||\langle 0002\rangle$
	−4.2	2.1	−80.8	−69.5

**Table 3:** The interplanar misfit between MgB_2_ {0002} and possible match planes of MB_2_ (M = Ti or Al) nucleation center (%).

MgB_2_/MB_2_	$\left\{0002\right\}|\left\{10\bar{1}1\right\}$	{0002}|{0002}	$\left\{0002\right\}|\left\{10\bar{1}0\right\}$	$\left\{0002\right\}|\left\{1\bar{2}10\right\}$
TiB_2_	−15.8	8.2	−49.2	13.9
AlB_2_	−15.5	7.6	−47.9	14.6

**Table 4:** The interatomic misfit along possible matching directions between MgB_2_ and MB_2_ (M = Ti or Al) nucleation center (%).

MgB_2_/MB_2_	$\langle 1\bar{2}10\rangle ||\langle 1\bar{2}10\rangle$	$\langle 1\bar{2}10\rangle ||\langle 10\bar{1}0\rangle$	$\langle 10\bar{1}0\rangle ||\langle 1\bar{2}10\rangle$	$\langle 10\bar{1}0\rangle ||\langle 10\bar{1}0\rangle$
TiB_2_	1.7	−70.3	43.3	1.7
AlB_2_	2.6	−68.7	43.8	2.6

## Data Availability

The data that support the findings of this study are openly available in KITOpen under https://publikationen.bibliothek.kit.edu/1000140178 (accessed on 1 January 2022).

## Supplementary Materials

The supporting information can be downloaded at: www.mdpi.com/article/10.3390/nano12111893/s1, Figure S1: The results of 2LiBH_4_-MgH_2_ without additives after desorption: STEM-HAADF image acquired from the corresponding position in Figure 2a and EDX elemental map of Mg; Figure S2: The results of 2LiBH_4_-MgH_2_ with 5 wt% 3TiCl_3_·AlCl_3_ after incomplete desorption: (a) STEM-HAADF image, and (b) the corresponding electron diffraction pattern; (c) STEM-HAADF image acquired from the selected area in (a) and (d) the corresponding EDX elemental map of Mg; Figure S3: The results of 2LiBH_4_-MgH_2_ with 0.625 mol% 3TiCl_3_·AlCl_3_ after desorption: (a) STEM-HAADF images showing the morphology of the generated MgB_2_ crystals; (b) electron diffraction pattern; (c) STEM-HAADF image acquired from the corresponding position in (a), and EDX map of Mg; Figure S4: The results of 2LiBH_4_-MgH_2_ with 20 mol% 3TiCl_3_·AlCl_3_ after desorption: STEM-HAADF image acquired from the selected position in Figure 3a, and the corresponding EDX elemental map of Mg, Ti, and Al; Figure S5: The results of 2LiBH_4_-MgH_2_ with 20 mol% 3TiCl_3_·AlCl_3_ after desorption: HRTEM image acquired from the position of purple agglomerates in Figure 4a, and the corresponding FFT, showing the existence of TiB_2_ (and AlB_2_); Figure S6: The results of 2LiBH_4_-MgH_2_ with 20 mol% 3TiCl_3_·AlCl_3_ after desorption: (a) STEM-HAADF image showing the distribution of MgB_2_ platelets; (b) electron diffraction patterns showing the crystallographic orientation of the corresponding MgB_2_ platelets indicated in (a); Figure S7: Simulated superimposed diffraction patterns of MgB_2_/Mg (a,b), and MgB_2_/TiB_2_ (c,d); Table S1: The interatomic misfit between ${\langle 0002\rangle }_{{\mathrm{MgB}}_{2}}$ and the possible matching directions of Mg nucleation center (%); Table S2: The interatomic misfit between ${\langle 0002\rangle }_{{\mathrm{MgB}}_{2}}$ and the possible matching directions of MB_2_ (M = Ti or Al) nucleation center (%).

## References

[1] Milanese C., Jensen T., Hauback B., Pistidda C., Dornheim M., Yang H., Lombardo L., Zuettel A., Filinchuk Y., Ngene P. (2019). Complex hydrides for energy storage. Int. J. Hydrog. Energy.

[2] Chen P., Xiong Z., Luo J., Lin J., Tan K.L. (2002). Interaction of hydrogen with metal nitrides and imides. Nature.

[3] Barkhordarian G., Klassen T., Dornheim M., Bormann R. (2007). Unexpected kinetic effect of MgB_2_ in reactive hydride composites containing complex borohydrides. J. Alloy. Compd..

[4] Vajo J.J., Salguero T.T., Gross A.F., Skeith S.L., Olson G.L. (2007). Thermodynamic destabilization and reaction kinetics in light metal hydride systems. J. Alloy. Compd..

[5] Hauback B.C., Detlef Stolten T.G. (2010). Task 22 of IEA HIA—Fundamental and Applied Hydrogen Storage Materials Development. 18th World Hydrogen Energy Conference 2010-WHEC 2010.

[6] Bösenberg U., Doppiu S., Mosegaard L., Barkhordarian G., Eigen N., Borgschulte A., Jensen T.R., Cerenius Y., Gutfleisch O., Klassen T. (2007). Hydrogen sorption properties of MgH_2_–LiBH_4_ composites. Acta Mater..

[7] Mauron P., Buchter F., Friedrichs O., Remhof A., Bielmann M., Zwicky C.N., Züttel A. (2008). Stability and reversibility of LiBH_4_. J. Phys. Chem. B.

[8] Orimo S.-I., Nakamori Y., Kitahara G., Miwa K., Ohba N., Towata S.-I., Züttel A. (2005). Dehydriding and rehydriding reactions of LiBH_4_. J. Alloy. Compd..

[9] Vajo J.J., Olson G.L. (2007). Hydrogen storage in destabilized chemical systems. Scr. Mater..

[10] Vajo J.J., Skeith S.L., Mertens F. (2005). Reversible storage of hydrogen in destabilized LiBH_4_. J. Phys. Chem. B.

[11] Bösenberg U., Kim J.W., Gosslar D., Eigen N., Jensen T.R., von Colbe J.B., Zhou Y., Dahms M., Kim D., Günther R. (2010). Role of additives in LiBH_4_–MgH_2_ reactive hydride composites for sorption kinetics. Acta Mater..

[12] Bösenberg U., Ravnsbæk D.B., Hagemann H., D’Anna V., Minella C.B., Pistidda C., van Beek W., Jensen T.R., Bormann R.d., Dornheim M. (2010). Pressure and temperature influence on the desorption pathway of the LiBH_4_− MgH_2_ composite system. J. Phys. Chem. C.

[13] Gross A.F., Vajo J.J., van Atta S.L., Olson G.L. (2008). Enhanced hydrogen storage kinetics of LiBH_4_ in nanoporous carbon scaffolds. J. Phys. Chem. C.

[14] Gosalawit–Utke R., Thiangviriya S., Javadian P., Laipple D., Pistidda C., Bergemann N., Horstmann C., Jensen T.R., Klassen T., Dornheim M. (2014). Effective nanoconfinement of 2LiBH_4_–MgH_2_ via simply MgH_2_ premilling for reversible hydrogen storages. Int. J. Hydrog. Energy.

[15] Huang X., Xiao X., Shao J., Zhai B., Fan X., Cheng C., Li S., Ge H., Wang Q., Chen L. (2016). Building robust architectures of carbon-wrapped transition metal nanoparticles for high catalytic enhancement of the 2LiBH_4_-MgH_2_ system for hydrogen storage cycling performance. Nanoscale.

[16] Deprez E., Justo A., Rojas T., López-Cartés C., Minella C.B., Bösenberg U., Dornheim M., Bormann R., Fernández A. (2010). Microstructural study of the LiBH_4_–MgH_2_ reactive hydride composite with and without Ti-isopropoxide additive. Acta Mater..

[17] Fan M.-Q., Sun L.-X., Zhang Y., Xu F., Zhang J., Chu H.-l. (2008). The catalytic effect of additive Nb2O5 on the reversible hydrogen storage performances of LiBH_4_–MgH_2_ composite. Int. J. Hydrog. Energy.

[18] Le T.-T., Pistidda C., Puszkiel J.n., Riglos M.a.V.C., Karimi F., Skibsted J., GharibDoust S.P., Richter B., Emmler T., Milanese C. (2018). Design of a nanometric AlTi additive for MgB_2_-based reactive hydride composites with superior kinetic properties. J. Phys. Chem. C.

[19] Kremer J.R., Mastronarde D.N., McIntosh J.R. (1996). Computer visualization of three-dimensional image data using IMOD. J. Struct. Biol..

[20] Gilbert P. (1972). Iterative methods for the three-dimensional reconstruction of an object from projections. J. Theor. Biol..

[21] Wold S., Esbensen K., Geladi P. (1987). Principal component analysis. Chemom. Intell. Lab. Syst..

[22] Abdi H., Williams L.J. (2010). Principal component analysis. Wiley Interdiscip. Rev. Comput. Stat..

[23] Lee S. (2003). Crystal growth of MgB_2_. Phys. C Supercond..

[24] Kong X., Wang Y., Li H., Duan X., Yu R., Li S., Li F., Jin C. (2002). Electron energy-loss spectroscopy characterization of the boron p-like density of states in MgB_2_. Appl. Phys. Lett..

[25] Deprez E., Munoz-Márquez M.A., Roldán M.A., Prestipino C., Palomares F.J., Minella C.B., Bosenberg U., Dornheim M., Bormann R., Fernández A. (2010). Oxidation state and local structure of Ti-based additives in the reactive hydride composite 2LiBH_4_+ MgH_2_. J. Phys. Chem. C.

[26] Fan X., Xiao X., Chen L., Wang X., Li S., Ge H., Wang Q. (2013). High catalytic efficiency of amorphous TiB_2_ and NbB_2_ nanoparticles for hydrogen storage using the 2LiBH_4_–MgH_2_ system. J. Mater. Chem. A.

[27] Zhang M.-X., Kelly P. (2005). Edge-to-edge matching model for predicting orientation relationships and habit planes—the improvements. Scr. Mater..

[28] Zhang M.-X., Kelly P. (2005). Edge-to-edge matching and its applications: Part II. Application to Mg–Al, Mg–Y and Mg–Mn alloys. Acta Mater..

[29] Zhang M.-X., Kelly P.M., Easton M.A., Taylor J.A. (2005). Crystallographic study of grain refinement in aluminum alloys using the edge-to-edge matching model. Acta Mater..

[30] Kelly P., Zhang M.-X. (2006). Edge-to-edge matching—The fundamentals. Metall. Mater. Trans. A.

[31] Yang J., Wang J., Wu Y., Wang L., Zhang H. (2007). Extended application of edge-to-edge matching model to HCP/HCP (α-Mg/MgZn_2_) system in magnesium alloys. Mater. Sci. Eng. A.

