# Supplementary material for: Microstructural Study of MgB2 in the LiBH4-MgH2 Composite by Using TEM
Source: Nanomaterials (Basel). 2022 May 31;12(11):1893. doi: 10.3390/nano12111893 (PMC9182164; doi:10.3390/nano12111893)
Supplement: Supplementary file 1 [file nanomaterials-12-01893-s001.zip › nanomaterials-1725863-supplementary.pdf]

nanomaterials

Microstructural Study of MgB_2 in the $LiBH_4$-MgH_2 Composite by Using TEM

Ou Jin [1,2], Yuanyuan Shang [3], Xiaohui Huang [2], Xiaoke Mu [2], Dorothée Vinga Szabó [1,2,4], Thi Thu Le [3], Stefan Wagner [1], Christian Kübel [2,4,5], Claudio Pistidda [3], and Astrid Pundt [1,*]

1 Institute of Applied Materials, Karlsruhe Institute of Technology, 76131 Karlsruhe, Germany; ou.jin@kit.edu (O.J.); dorothee.szabo@kit.edu (D.V.S.); stefan.wagner3@kit.edu (S.W.)
2 Institute of Nanotechnology, Karlsruhe Institute of Technology, 76344 Eggenstein-Leopoldshafen, Germany; xiaohui.huang@partner.kit.edu (X.H.); xiaoke.mu@kit.edu (X.M.); christian.kuebel@kit.edu (C.K.)
3 Institute of Hydrogen Technology, Helmholtz-Zentrum hereon GmbH, 21502 Geesthacht, Germany; yuanyuan.shang@hzg.de (Y.S.); thi.le@hzg.de (T.T.L.); claudio.pistidda@hzg.de (C.P.)
4 Karlsruhe Nano Micro Facility, Karlsruhe Institute of Technology, 76344 Eggenstein-Leopoldshafen, Germany
5 Joint Research Laboratory Nanomaterials, Technical University of Darmstadt, 64206 Darmstadt, Germany
* Correspondence: astrid.pundt@kit.edu; Tel: +49-721-608-42345

Keywords: hydrogen storage; transmission electron microscopy; crystallography; reactive hydride composite; additive

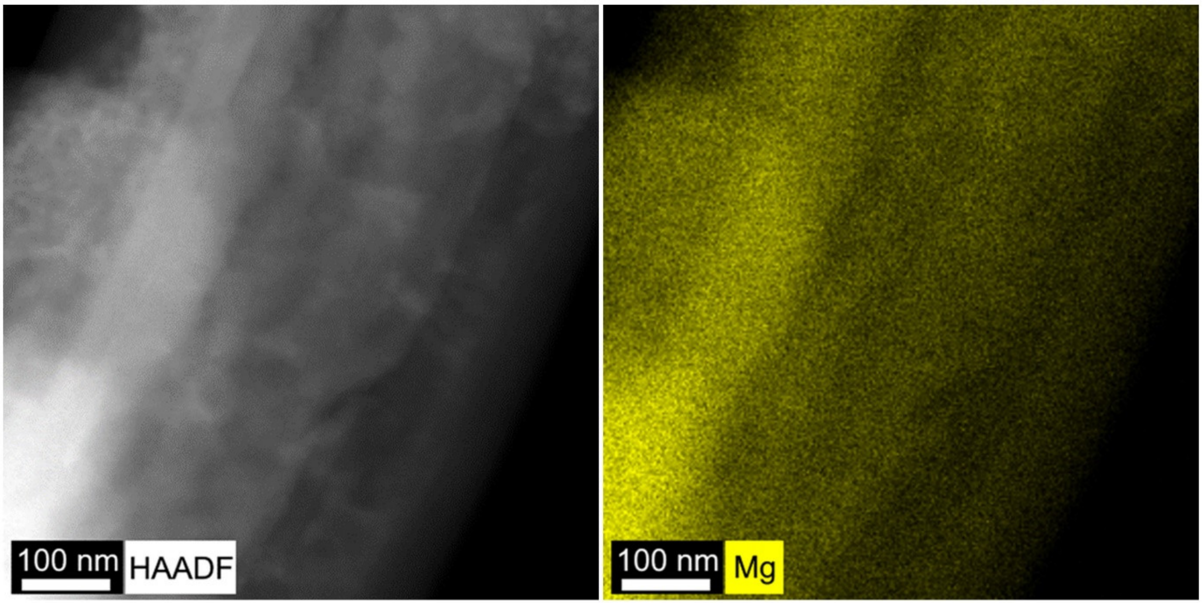

Figure S1. The results of $2LiBH_4$-MgH_2 without additives after desorption: STEM-HAADF image acquired from the corresponding position in Figure 2a and EDX elemental map of Mg.

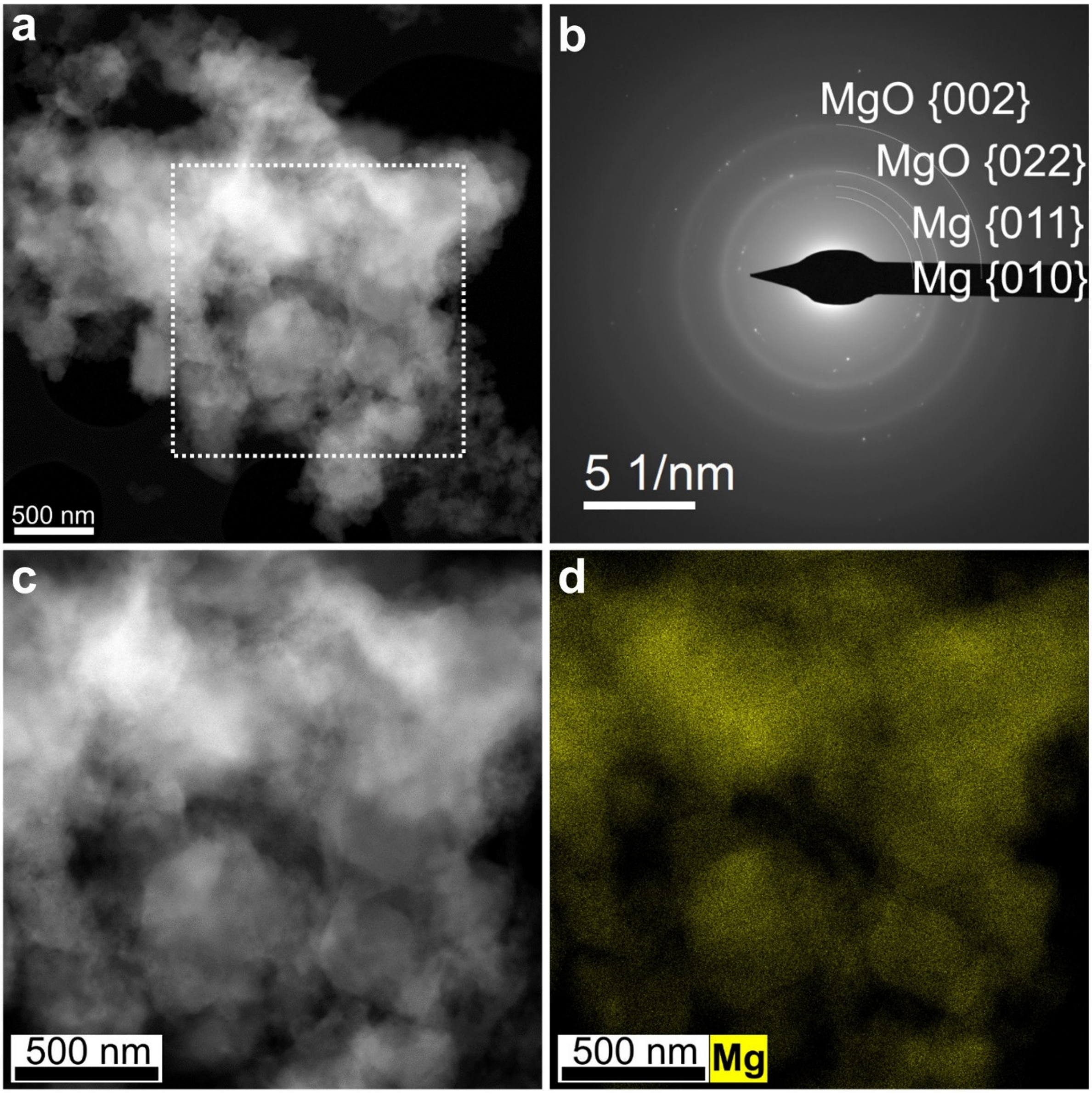

Figure S2. The results of $2LiBH_4$-MgH_2 with 5 wt% $3TiCl_3 \cdot AlCl_3$ after incomplete desorption: (**a**) STEM-HAADF image, and (**b**) the corresponding electron diffraction pattern; (**c**) STEM-HAADF image acquired from the selected area in (**a**) and (**d**) the corresponding EDX elemental map of Mg.

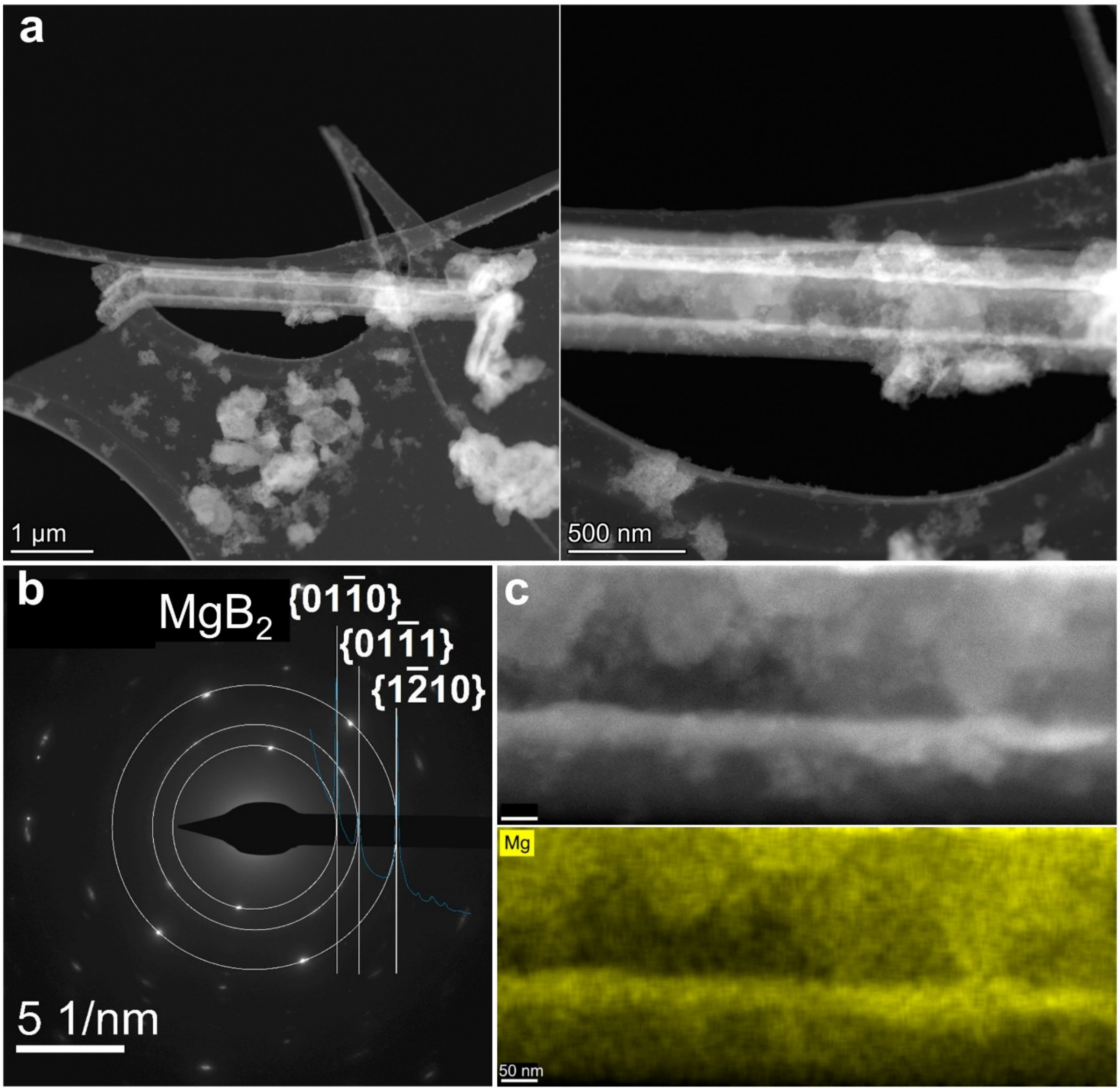

Figure S3. The results of $2LiBH_4$-MgH_2 with 0.625 mol% $3TiCl_3{\cdot}AlCl_3$ after desorption: (**a**) STEM-HAADF images showing the morphology of the generated MgB_2 crystals; (**b**) electron diffraction pattern; (**c**) STEM-HAADF image acquired from the corresponding position in (**a**), and EDX map of Mg.

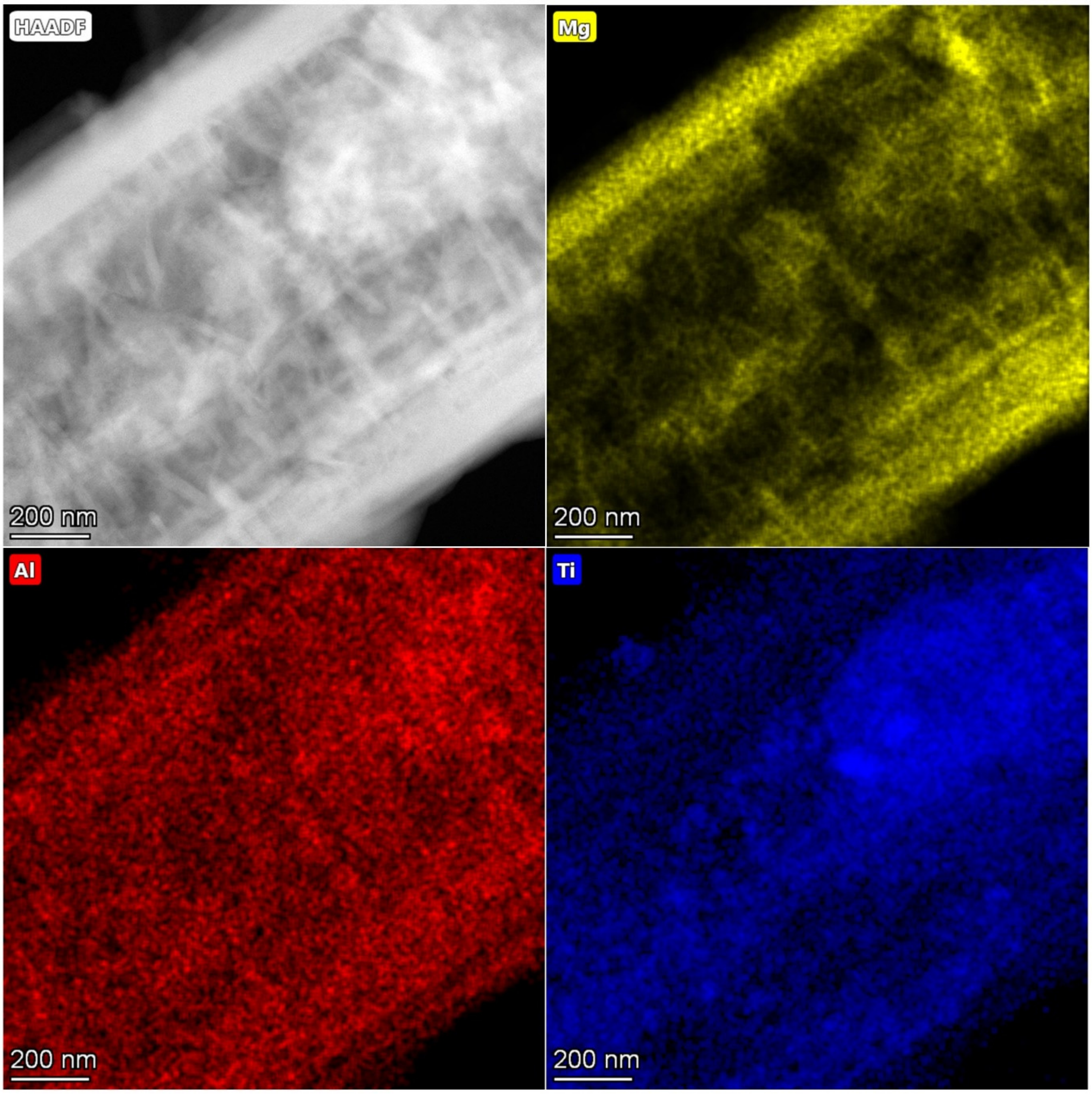

Figure S4. The results of $2LiBH_4$-MgH_2 with 20 mol% $3TiCl_3{\cdot}AlCl_3$ after desorption: STEM-HAADF image acquired from the selected position in Figure 3a, and the corresponding EDX elemental map of Mg, Ti, and Al.

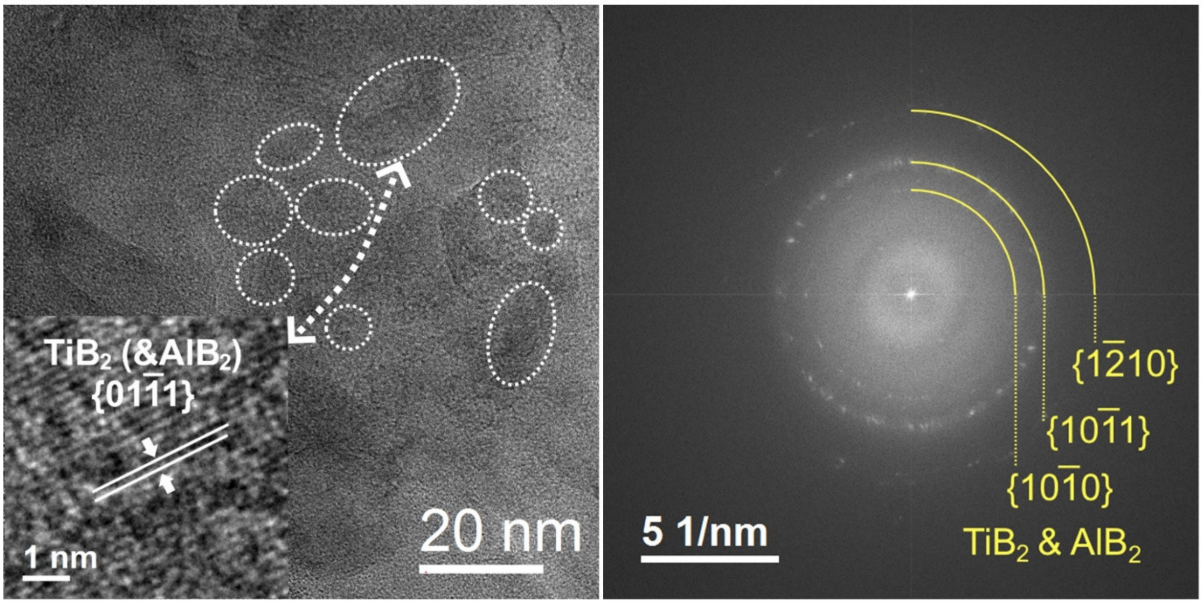

Figure S5. The results of 2LiBH$_4$-MgH$_2$ with 20 mol% 3TiCl$_3$·AlCl$_3$ after desorption: HRTEM image acquired from the position of purple agglomerates in Figure 4a, and the corresponding FFT, showing the existence of TiB$_2$ (and AlB$_2$).

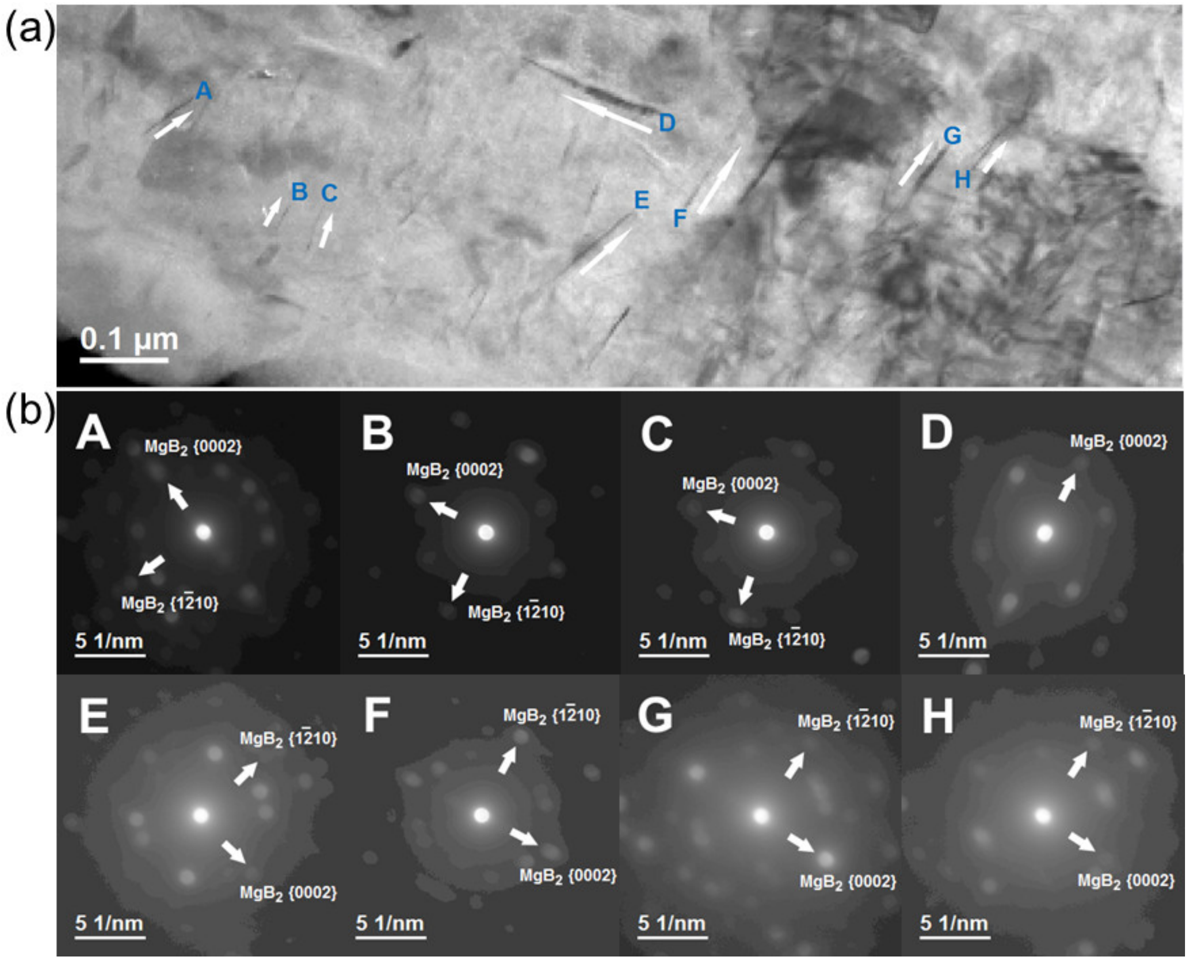

Figure S6. The results of 2LiBH$_4$-MgH$_2$ with 20 mol% 3TiCl$_3$·AlCl$_3$ after desorption: (**a**) STEM-HAADF image showing the distribution of MgB$_2$ platelets; (**b**) electron diffraction patterns showing the crystallographic orientation of the corresponding MgB$_2$ platelets indicated in (**a**).

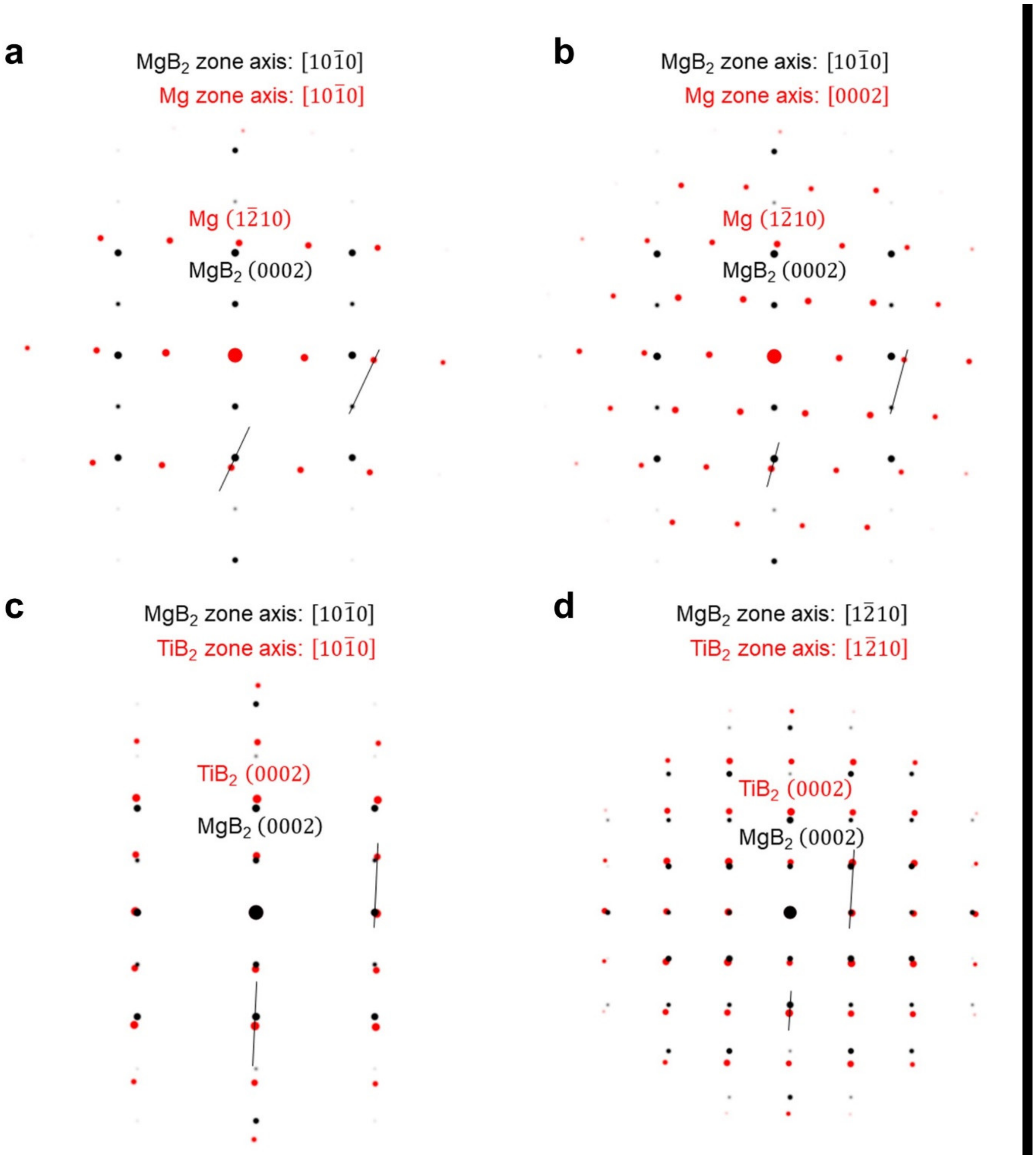

Figure S7. Simulated superimposed diffraction patterns of MgB_2 / Mg (**a**–**b**), and MgB_2 / TiB_2 (**c**–**d**).

Table S1. The interatomic misfit between $\langle 0002\rangle_{MgB_2}$ and the possible matching directions of Mg nucleation center (%).

MgB_2/Mg	$\langle 0002\rangle \| \langle 10\bar{1}0\rangle$	$\langle 0002\rangle \| \langle 0002\rangle$	$\langle 0002\rangle \| \langle 1\bar{2}10\rangle$
	48.6	58.4	8.5

Table S2. The interatomic misfit between $\langle 0002\rangle_{MgB_2}$ and the possible matching directions of MB_2 (M = Ti or Al) nucleation center (%).

MB_2/Mg	$\langle 0002\rangle \| \langle 10\bar{1}0\rangle$	$\langle 0002\rangle \| \langle 0002\rangle$	$\langle 0002\rangle \| \langle 1\bar{2}10\rangle$
TiB_2	-49.2	8.2	13.9
AlB_2	-47.9	7.6	14.6
